# Authors' Reply

**DOI:** 10.1371/journal.pmed.0020085

**Published:** 2005-03-29

**Authors:** Sam Gandy, Suzana Petanceska

**Affiliations:** Farber Institute for Neurosciences, Thomas Jefferson UniversityPhiladelphia, PennsylvaniaUnited States of America; Nathan Kline Institute at New York UniversityOrangeburg, New YorkUnited States of America

We appreciate the note from Drs. Koudinov and Berezov [1]. In our opinion, no model has yet been presented that plausibly accounts for all the data on statins, cholesterol, amyloid-ß protein (Aß), and Alzheimer disease. In our paper [2], we present evidence that the isoprenoid pathway contributes to statin-activated shedding of the APP ectodomain in cultured cells. We do not yet know which (if any) other “cholesterol-related” Alzheimer phenomena are also attributable to modulation of isoprenoids, Rho, or ROCK.

Previously, conventional wisdom held that Aß load and hypercholesterolemia were directly related, based on observations that high-fat diet aggravated amyloid pathology in plaque-forming mice [3,4,5]. More recently, however, the formulation that statins act simply via cholesterol-lowering fails to account for several observations that cannot immediately be reconciled, either with the original “dogma” or with each other.

First, Fagan et al. [6] questioned the role of cholesterol as the final common pathway in Aß load specification, since, in their experiments, low cholesterol per se apparently had no impact on brain Aß load in plaque-forming transgenic mice. Then, equally puzzling pharmacological data emerged. Atorvastatin was shown to lower brain amyloid load and Aß levels, but brain cholesterol levels were unaffected by the drug [7]. In an apparent complete contradiction with the original observations, now, some investigators have been able to devise circumstances under which there is an inverse relationship between cholesterol and Aß, with low neuronal cholesterol increasing Aß generation [8], and vice versa [9]. These newer observations are unexpected and extremely puzzling, and no comprehensive explanation has yet emerged.

For those readers seeking an update on this challenging area, we would direct your attention to the Alzheimer Research Forum Web page (http://www.alzforum.org/new/detailprint.asp?id=1135), where you will find an excellent review of the literature as well as a series of evaluations of how our data fit into existing scenarios and models regarding cholesterol, statins, cerebral amyloidosis, and the cognitive failure of Alzheimer disease.
